# Health Care Professionals’ Experiences and Perspectives on Using Telehealth for Home-based Palliative Care: Scoping Review

**DOI:** 10.2196/43429

**Published:** 2023-03-29

**Authors:** Elias David Lundereng, Andréa Aparecida Gonçalves Nes, Heidi Holmen, Anette Winger, Hilde Thygesen, Nina Jøranson, Christine Råheim Borge, Olav Dajani, Kari L Mariussen, Simen A Steindal

**Affiliations:** 1 Lovisenberg Diaconal University College Oslo Norway; 2 European Palliative Care Research Centre (PRC) Department of Oncology Oslo University Hospital Oslo Norway; 3 Faculty of Health Sciences Department of Nursing and Health Promotion Oslo Metropolitan University Oslo Norway; 4 The Intervention Centre Oslo University Hospital Oslo Norway; 5 Faculty of Health Studies VID Specialized University Oslo Norway; 6 Department of Occupational Therapy Prosthetics and Orthotics Oslo Metropolitan University Oslo Norway; 7 Research Department Lovisenberg Diaconal Hospital Oslo Norway; 8 Department of Interdisciplinary Health Sciences University of Oslo Oslo Norway

**Keywords:** health technology, homecare services, palliative care, review, telehealth, telemedicine, care, technology, feasibility, data, decision-making, policy makers

## Abstract

**Background:**

Telehealth seems feasible for use in home-based palliative care (HBPC). It may improve access to health care professionals (HCPs) at patients’ homes, reduce hospital admissions, enhance patients’ feelings of security and safety, and increase the time spent at home for patients in HBPC. HBPC requires the involvement of various HCPs such as nurses, physicians, allied health professionals, dietitians, psychologists, religious counselors, and social workers. Acceptance of the use of technology among HCPs is essential for the successful delivery of telehealth in practice. No scoping review has mapped the experiences and perspectives of HCPs regarding the use of telehealth in HBPC.

**Objective:**

The aim of this review was to systematically map published studies on HCPs’ experiences and perspectives on the use of telehealth in HBPC.

**Methods:**

A scoping review was conducted using the methodology of Arksey and O’Malley. The review was reported according to the Preferred Reporting Items for Systematic Reviews and Meta-Analyses extension for scoping reviews. A systematic search was performed in AMED, CINAHL, Embase, MEDLINE, PsycINFO, and Web of Science for studies published in peer-reviewed journals between January 1, 2000, and August 23, 2022. The reference lists of the included papers were hand searched to identify additional studies. The inclusion criteria were (1) studies using qualitative, quantitative, or mixed methods; (2) studies including HCPs using telehealth with patients in HBPC; (3) studies on HCPs’ experiences and perspectives on the use of telehealth in HBPC; (4) studies published between January 1, 2000, and August 23, 2022; and (5) studies published in English, Portuguese, Norwegian, Danish, Swedish, or Spanish. Pairs of authors independently included studies and extracted data. The first 2 stages of thematic synthesis were used to thematically organize the data.

**Results:**

This scoping review included 29 papers from 28 studies. Four descriptive themes were identified: (1) easy to use but technological issues undermine confidence, (2) adds value but personal and organizational barriers challenge adoption, (3) potential to provide useful and meaningful patient-reported data, and (4) mutual trust as a prerequisite for interpersonal relationships.

**Conclusions:**

Telehealth in HBPC seems to be easy to use and may improve the coordination of care, time efficiency, clinical assessments, and help build and enhance personal and professional relationships. However, the introduction of technology in HBPC is complex, as it may not align well with the overall aim of palliative care from HCPs’ point of view. Further, changes in practice and requirements for HCPs may reduce motivation for the use of telehealth in HBPC. HCPs consider themselves to have central roles in implementing telehealth, and a lack of acceptance and motivation is a key barrier to telehealth adoption. Policy makers and telehealth developers should be aware of this potential barrier when developing or implementing new technology for use in HBPC.

**International Registered Report Identifier (IRRID):**

RR2-10.2196/33305

## Introduction

A key goal in palliative care is to provide access to coordinated, continuous, and specialized palliative care services at the location desired by patients [1]. Palliative care is a multidisciplinary approach and requires the involvement of various health care professionals (HCPs) such as nurses, physicians of different specialties (eg, general practitioners, palliative physicians, anesthetists, psychiatrists, oncologists, and other disease-specific specialists), allied health professionals (eg, physiotherapists, occupational therapists, speech, and language therapists), dietitians, psychologists, religious counselors, and social workers [2]. The preferred place of care for most palliative care patients is their own homes [3,4]. However, unmet palliative care needs, uncoordinated care, and insufficient communication with HCPs [5,6], as well as the demanding collaboration between specialists and home care professionals, make this challenging [7]. The increasing health care costs in the final years of life [8] are primarily driven by hospitalizations [9,10]. Consequently, switching from hospital-oriented palliative care to community-based palliative care has become a priority for health care systems to reduce the societal costs of the aging population [8,11].

Telehealth is defined “as the provision of health care remotely by means of a variety of telecommunication tools” [12]. The adoption of telehealth is rapidly changing the way we deliver health care, and the use of electronic health records, decision support tools, and videoconferencing has already been implemented in many countries [13]. The use of telehealth in home-based palliative care (HBPC) may enhance access to HCPs at home, promote self-monitoring, and enhance patients’ feelings of safety and security [14]. Telehealth may contribute to cost-effective palliative care by preventing and reducing hospital admissions, emergency department attendance, and hospital deaths [15-17]. It may also facilitate collaboration between different health care services by improving information flow [17,18]. During the COVID-19 pandemic, the use of telehealth in palliative care was promoted as a way to improve communication between isolated patients and their families, and between patients and HCPs, while reducing the risk of exposing vulnerable patients to hospital-based pathogens [19-21].

While telehealth appears promising in delivering HBPC, many HCPs feel that telehealth is unsuited for the palliative care population because of patients’ rapid deterioration, age, and illness burden [16]. HCPs may perceive palliative care as *high touch* rather than *high tech*, and they could be concerned about telehealth being burdensome for patients [22,23]. There is also a concern that the increasing amount of patient-generated data makes HCPs more attentive to the technology than to the patient, at the expense of actual support and caregiving. This could be particularly detrimental in a palliative care context in which a trusting relationship is a key factor [13].

A lack of acceptance of using telehealth among HCPs seems to be a barrier to implementing telehealth in HBPC [9]. Telehealth studies must identify the barriers to and facilitators of the adoption of technology, as these requirements will influence the design, use, and function of the developed technology [24]. Previous literature reviews regarding the use of telehealth in palliative care have primarily focused on pediatric palliative care [25,26], older patients with chronic conditions [22,27,28], or patients with cancer [29-31] and have examined patient or caregiver outcomes and experiences [14,17,23,32]. Some systematic reviews have investigated the use of video consultations only [33] or of technology in general and specialized palliative care from multiple perspectives, such as those of patients, caregivers, and HCPs [34]. There have also been systematic reviews regarding how telehealth can improve access to and the extension of palliative care services in rural areas [35,36].

With the rapid implementation of telehealth in HBPC and the emerging research in this field, there is a need to describe findings and studies related to HCPs’ experiences with the use of telehealth. Although technology acceptance among HCPs is essential for the successful implementation of telehealth in HBPC, initial literature searches showed that no scoping review has examined the experiences and perspectives of HCPs on the use of telehealth in HBPC. A scoping review is suitable for gathering literature in disciplines with emerging evidence [37], for helping identify research gaps regarding telehealth in HBPC associated with HCPs, and for determining the feasibility of conducting a systematic review [38]. Consequently, the aim of this scoping review was to systematically map published studies on the use of telehealth in HBPC, with a focus on the experiences and perspectives of HCPs. Our research question was as follows: what is known from published studies about HCPs’ experiences and perspectives on using telehealth in HBPC?

## Methods

### Overview

This scoping review used the methodology of Arksey and O’Malley [38], which consists of the following stages: (1) identifying the research question; (2) identifying relevant studies; (3) selecting studies; (4) charting the data; and (5) collating, summarizing, and reporting the results. The reporting of this scoping review was guided by the Preferred Reporting Items for Systematic Reviews and Meta-Analyses (PRISMA) extension for scoping reviews (PRISMA-ScR) [39]. The PRISMA-ScR checklist is provided in Multimedia Appendix 1. Deviations from the published protocol [40] are shown in Multimedia Appendix 2.

### Eligibility Criteria

The inclusion and exclusion criteria are shown in Textbox 1. The first and the last authors independently tested the inclusion and exclusion criteria on the same 5% of the retrieved studies to assess the robustness of the criteria in capturing relevant publications. The language criteria are based on the authors’ fluency in the included languages.

**Inclusion and exclusion criteria.**

**Type of studies**

- Inclusion
  Qualitative, quantitative, or mixed methods studies published in peer-reviewed journals
- Exclusion
  Any type of review, case report, letter, book chapter, guideline, comment, discussion, editorial, conference abstract, study protocol, master’s thesis, or PhD thesis

**Time period**

- Inclusion
  January 1, 2000, to August 23, 2022
- Exclusion
  Before January 1, 2000, and after August 23, 2022

**Language criteria**

- Inclusion
  English, Portuguese, Norwegian Danish, Swedish, or Spanish
- Exclusion
  All other languages

**Type of participants**

- Inclusion
  Papers including health care professionals using telehealth with patients in home-based palliative care
- Exclusion
  Papers including health care professionals using telehealth with patients outside of a palliative care environment, those that only tend to family caregivers, or studies that do not present data from the perspective of health care professionals

**Phenomenon of interest**

- Inclusion
  Health care professionals’ experiences of and perspectives on the use of telehealth in home-based palliative care
- Exclusion
  Health care professionals’ experiences of and perspectives on the use of telehealth at home without interaction with the patient, or experience of use of telehealth in a hospital, nursing home, or hospice. Telehealth includes only telephone follow-up

### Information Sources

A systematic search was conducted in the electronic databases of AMED, CINAHL, Embase, MEDLINE, PsycINFO, and Web of Science on July 5, 2021. The search was updated on August 23, 2022.

### Search Strategy

The search strategy in MEDLINE was developed by an experienced research librarian (KM) and by the first and the last authors using MeSH terms and text words related to three main themes: (1) palliative care, (2) telehealth, and (3) home setting. The search strategy was piloted to validate the appropriateness of text words and MeSH terms, and it was peer-reviewed by a second experienced research librarian (MAØ) using the Peer Review of Electronic Search Strategies checklist [41]. The search strategy was adapted to each database (Multimedia Appendix 3). The reference lists of included papers were hand searched to identify additional studies of relevance.

### Data Management

The research librarian uploaded the publications identified in the searches to EndNote for the removal of duplicates and transferred the publications into the web application Covidence [42] to facilitate the storage and independent selection of eligible publications.

### Selection Process

Pairs of authors independently screened titles, abstracts, and full-text papers to determine their eligibility. Conflicts among the pairs were resolved by the first and the last authors based on discussions and consensus.

### Data Collection Process

A standardized data charting form was developed and used to chart relevant data from the included papers. The data charting form was reviewed by the entire research team prior to the data collection and was pilot tested by the first and the last authors on 5 studies to ensure that the form captured the information accurately. The following data were included: authors, publication year, country, aim, sample, telehealth solution, design, and findings related to the research question. Pairs of authors conducted the data charting. One author extracted the data, while the other author controlled for accuracy. Any discrepancies were discussed among the pairs of authors, and agreement was based on consensus or the involvement of the first and the last authors.

### Risk of Bias and Quality Appraisal

The sources of evidence included in this review were not assessed for risk of bias or methodological quality as scoping reviews aim to provide an overview of the existing literature regardless of methodological rigor or risk of bias [39].

### Data Synthesis

**Figure 1:**
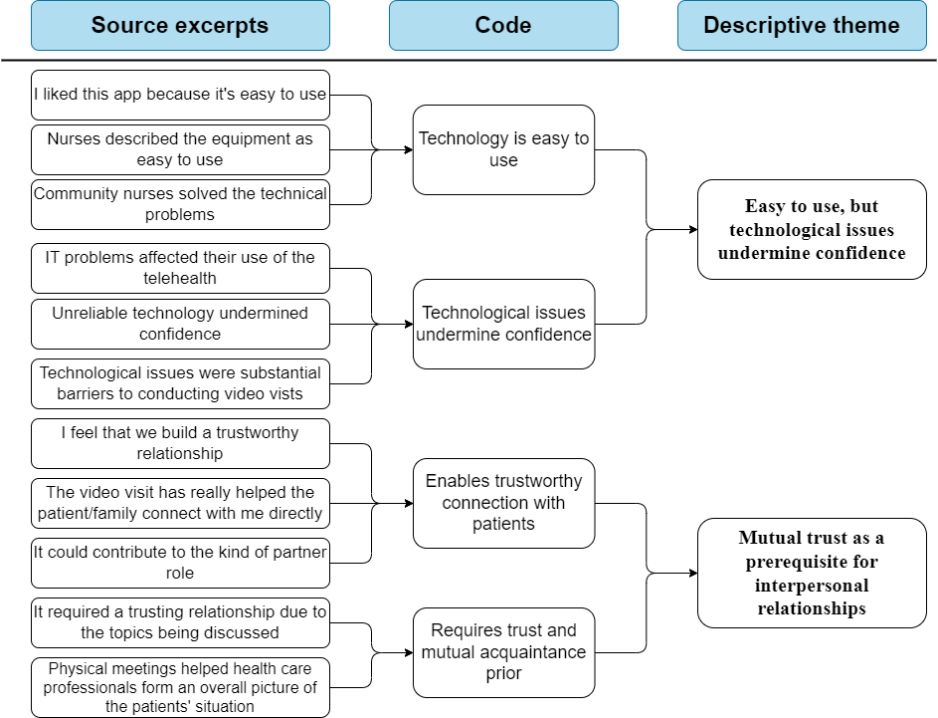
Example of a hierarchical coding tree for 2 descriptive themes.

The first 2 stages of thematic synthesis [43] were used to inductively organize the data. The qualitative data analysis software NVivo (QSR International) [44] was used to organize the data. In stage 1 of the thematic synthesis, the data from the results section of the studies included were read multiple times and coded line by line by the first author to identify patterns, similarities, and differences in the experiences and perspectives of HCPs on the use of various technological solutions in HBPC. Numerical data presented in tables and figures were transformed into a qualitative format [45]. The line-by-line coding resulted in 303 source excerpts across all studies included. In stage 2, the excerpts were compared for similarities and differences, and they were merged and organized into 25 codes. The codes were then organized into 4 descriptive themes using a low degree of abstraction and interpretation to develop descriptions grounded in the included material that answered the aim of the scoping review. The codes and descriptive themes were discussed with the last author, and all the authors agreed on the final descriptive themes. This enhanced the trustworthiness of the findings, as the members of the research team have diverse clinical and research expertise. To further illustrate the process of organizing the data [46], an example of a hierarchical coding tree for 2 descriptive themes is illustrated in Figure 1. A frequency table illustrating which papers were included in which descriptive themes was made (Table 1).

**Table 1:** Articles included in the thematic groupings.

Theme	Study	Articles, n
Easy to use but technological issues undermine confidence	Osuji et al [47], Nguyen et al [48], Funderskov et al [49], Hochstenbach et al [50], Lind et al [51], Harding et al [52], Whitten et al [53], Adam et al [54], Stern et al [55], Whitten et al [56], Shulver et al [57], Miyazaki et al [58], Read Paul et al [59], Collier et al [60], McCall et al [61], Oelschlägel et al [62], Bhargava et al [63], Cameron et al [64], Eastman et al [65], Haydon et al [66], Scofano et al [67], and Weck et al [68]	22
Adds value but personal and organizational barriers challenge adoption	Bonsignore et al [69], Collier et al [60], Funderskov et al [49] Hackett et al [70], Helleman et al [71], Oelschlägel et al [62], Read Paul et al [59], Shulver et al [57], Harding et al [52], Whitten et al [56], Hochstenbach et al [50], Lind et al [51], Nguyen et al [48], van Gurp et al [72], Stern et al [55], Haydon et al [66], Scofano et al [67], and Weck et al [68]	18
Potential to provide useful and meaningful patient-reported data	Collier et al [60], Hackett et al [70], Harding et al [52], Hochstenbach et al [50], Lind et al [51], McCall et al [61], Adam et al [54], Funderskov et al [49], Read Paul et al [59], Whitten et al [53], Miyazaki et al [58], Tieman et al [73], van Gurp et al [74], Nguyen et al [48], Whitten et al [56], Oelschlägel et al [62], Shulver et al [57], Alodhayani et al [75], Bhargava et al [63], Cameron et al [64], Haydon et al [66], Scofano et al [67], and Weck et al [68]	23
Mutual trust as a prerequisite for interpersonal relationships	Bonsignore et al [69], Funderskov et al [49], Helleman et al [71], Hochstenbach et al [50], Lind et al [51], McCall et al [61], Nguyen et al [48], Oelschlägel et al [62], van Gurp et al [72], Whitten et al [56], Collier et al [60], Miyazaki et al [58], Tieman et al [73], Hackett et al [70], van Gurp et al [74], Osuji et al [47], Alodhayani et al [75], Cameron et al [64], Eastman et al [65], Haydon et al [66], and Scofano et al [67]	21

## Results

### Overview

**Figure 2:**
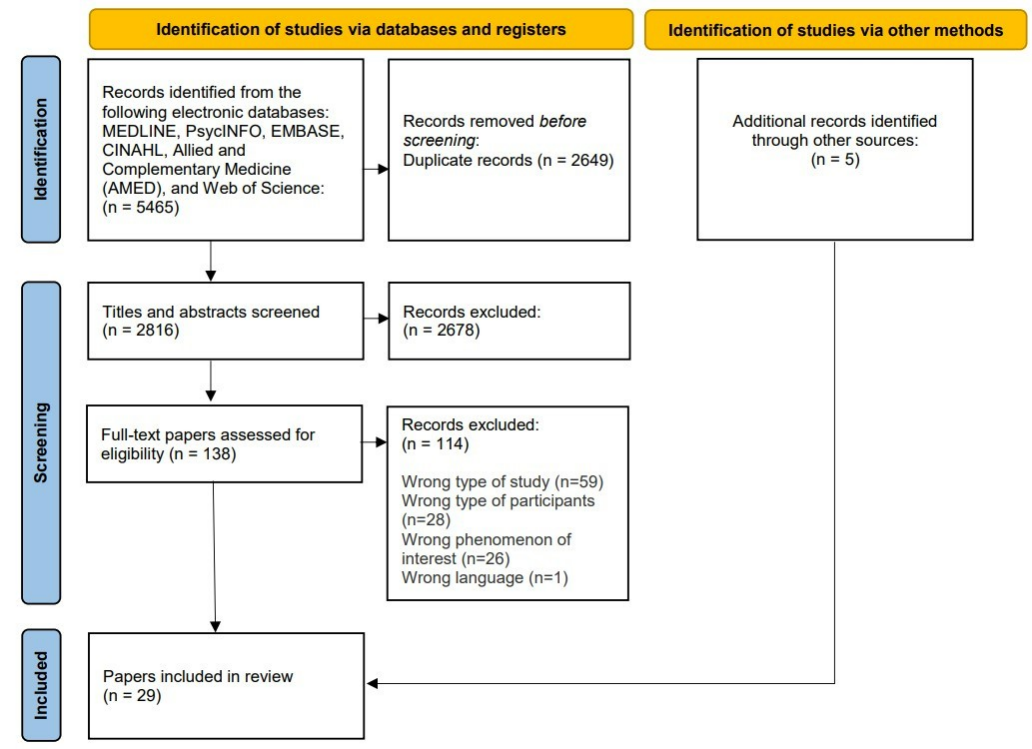
Preferred Reporting Items for Systematic Reviews and Meta-Analyses flowchart.

The search yielded 5465 citations. After the removal of 2649 duplicates, 2816 citations were screened. The full texts of 138 citations were read; 114 citations were excluded. Five additional citations were identified through other sources, such as hand searches and citation searching. A total of 29 papers from 28 studies were included. The reason for the exclusion of full-text papers is shown in Figure 2.

### Description of the Studies Included

The studies included were conducted in the United States (n=6), Australia (n=5), the Netherlands (n = 4), Canada (n=4), the United Kingdom (n=3), Germany (n=1), Denmark (n=1), Brazil (n=1), Saudi Arabia (n=1), Sweden (n=1), Norway (n=1), and cross-national (India, Uganda, and Zimbabwe, n=1). The sample size of the studies included ranged from 4 to 111 participants, and in 12 papers, the samples consisted of fewer than 10 participants. In all but 1 [68] study, the samples consisted of nurses with various specialties [47-67,69-75]; in 13 studies, physicians were included [47,49,51,52,54,63,64,66,68, 70,72,74,75]; and in 3 studies, hospice workers (ie, nurses, physicians, social workers, and spiritual care providers) were included [53,56,64]. Eleven studies had multiprofessional perspectives, including HCPs within rehabilitation, allied health, residential care, and palliative care [57,66]; case managers, coordinators, and respiratory therapists [58]; physician consultants [59]; telehealth providers or technologists [69,75]; and rehabilitation physicians, occupational therapists, physical therapists, speech therapists, dieticians, and social workers [49,62,71]. Three studies included only nurses in their sample [48,67,73], while 1 study included only physicians [68].

In the majority of the studies (n=13), patients with cancer receiving palliative care were the recipients of the telehealth intervention [49-51,54,55,59,61-63,70,72-74]. Eleven studies did not report specific diagnoses other than serious illnesses [47,48,57,58] or patients receiving palliative care [60,65,66,69] or end-of-life care [53,56,64]. One study included patients with amyotrophic lateral sclerosis [71], 1 study included patients with major organ failure or HIV/AIDS in addition to patients with cancer [52], 1 study included geriatric patients [75], 1 study included patients with renal disease receiving home dialysis [67], and 1 study included patients with neurological disease or diseases with neurological symptoms [68].

Thirteen papers used a qualitative design [49-51,53,54,57,60,62,66,70,72,74,75], 12 papers used a mixed or multimethods design [47,52,55,56,58,59,61,64,67-69,71], and 4 papers used a quantitative design [48,63,65,73]. The characteristics of the studies included are shown in Multimedia Appendix 4.

Video-based technology (n=16) was the most frequently used telehealth app in HBPC [47-49,53,56-59,64-68,72-74]. Teleconsultations among patients, families, and HCPs were used to discuss patients’ needs, concerns, symptoms, and other problems and to give patients and their families comfort and advice. In 7 studies, telehealth was delivered by hospital-based HCPs [49,57,66,67,72-74]; in 3 studies, telehealth was delivered by hospice workers [53,56,64]; and in 6 studies, telehealth was delivered by home care professionals [58,65] or by home care professionals in collaboration with hospital-based staff [47,48,59,68].

Web-based apps (n=12) intended for use on mobile phones [50,52,55,61,75], tablets [54,60,62,69,71], or personal computers [63,70], as well as digital pens and diaries [51] (n=1), were used for pain education and for monitoring and managing pain and other symptoms. Four studies applied combinations of video-based technology for conferencing and patient-reported data or monitoring [55,60,69,75]. In 7 studies, the telehealth delivery was hospital based [50,51,55,61,63,71,75]; in 5 studies, it was community based [54,60,62,69,70]; and 1 study had a combination of community- and hospital-based telehealth delivery [52].

We identified four descriptive themes from the studies included: (1) easy to use but technological issues undermine confidence, (2) adds value but personal and organizational barriers challenge adoption, (3) potential to provide useful and meaningful patient-reported data, and (4) mutual trust as a prerequisite for interpersonal relationships.

### Easy to Use but Technological Issues Undermine Confidence

Across studies, HCPs expressed that telehealth was acceptable, comfortable, interesting, and easy to learn and use. In most cases, they did not perceive the use of telehealth as burdensome, time consuming, or onerous. Rather, they were enthusiastic about new technologies and felt competent in troubleshooting technical problems [47-56,64,67,68]. HCPs considered that they had sufficient skills to perform key palliative care activities in the context of telehealth, such as video visits [47,57,58,68]. They also reported that patients and families found telehealth easy to operate and useful for improving their access to help and providing comfort and enhanced feelings of safety compared with in-person care [48,50,51,61,63,64,66,68].

Equipment problems, such as unreliable, slow-running, or crashing technology [48,54,55,63,65]; issues with the screen size [54,68]; a lack of internet connectivity [47,48,52,59,64,68]; and audio or imaging issues [58,59,68] were reported. Unreliable technology and connectivity issues undermined HCPs’ confidence in using telehealth, and they felt personally and professionally responsible when telehealth solutions failed [48,60]. Furthermore, a lack of functionalities, such as tailored, personalized, or supplementary questions for symptom assessments [50,54,61,62], retrospective logging of breakthrough doses or other patient data [51,54], upload confirmation [52], chat functionality [64], and equipment portability [55], were reported. A lack of desired functionality increased the likelihood of HCPs reverting to former ways of working [48,60]. They also expressed concerns regarding data security, lack of privacy during video consultations, and the legality of digital patient assessments [48,49,58-60].

### Adds Value but Personal and Organizational Barriers Challenge Adoption

HCPs reported that telehealth added value to HBPC, as it improved their access to patients, facilitated quick and timely responses, and improved time efficiency, quality, coordination, and continuity of care without increasing their overall workload compared with in-person visits [49,59,60,62,66,68-71,76]. For rural HCPs, an added benefit was that video visits increased the capacity and extension of palliative care services while minimizing the need for traveling [57,60,66,69]. Furthermore, telehealth provided an avenue or entry point to follow-up on isolated patients or patients who were reluctant to let HCPs into their homes [57,62].

Telehealth brought with it new tasks, different responsibilities, and unknown technologies, which were reported as challenging to adjust to [50-53,56,57,60]. HCPs were not always motivated to use new technology, and a lack of knowledge, understanding, and familiarity with telehealth reduced their engagement in using it as intended or in encouraging patients to use it [48,50,51,53,56,60,70]. However, prior experience with the use of technology, either through telehealth or with the use of technological devices, such as tablets, in daily clinical practice enhanced the acceptance of and confidence in using telehealth [49,57,67]. A lack of available comprehensive technical support [53,57] and integration with existing health care services were factors that negatively affected the successful adoption of telehealth services [50,55,57,62,72]. Proactive support and motivation from telehealth researchers or HCPs delivering telehealth increased encouragement among HCPs [53,62,70].

### Potential to Provide Useful and Meaningful Patient-Reported Data

Overall, HCPs perceived telehealth to provide meaningful, timely, synchronous, and asynchronous patient-reported clinical data. The data supported clinical assessments and mutual decision-making between patients and HCPs, improved HCPs’ assessment and understanding of patients’ symptoms, and enhanced symptom control [50-52,54,60,61,68,70]. Telehealth made HCPs more responsive and attentive to changes in patients’ symptoms, and it made patient-reported symptom assessments more actively used in decision-making [49,51,53,54,59,61,63,66,67,70]. The patient-generated data helped HCPs prioritize patients based on the needs of the patients [52,58,70]. Moreover, the visual features of telehealth enabled HCPs to remotely notice visual cues of deterioration, such as facial color and the patients’ surroundings, or to assess the patients’ living situations and emotional states [49,54,58-60,64,68,73-75]. Video visits made it possible to assist other HCPs or families who were present with the patient in doing clinical assessments, provide training in using medical equipment, or coordinate during an emergency [48,53,56,58,64,75].

Although telehealth offered useful insights into patients’ conditions, HCPs were sometimes concerned about missing important patient information. They expressed that video technology did not adequately convey important or smaller nuances of visible cues, such as body language, facial expressions, physical signs of decline, and living situations [57,62,70]. Moreover, clinical data obtained from patient reporting were sometimes perceived as ambiguous and dependent on HCPs’ experiences and knowledge of the individual patient, leading to different responses from different HCPs [50,51,60,62]. One study described that for patients with cognitive impairment, patient information was primarily conveyed through the families, creating uncertainty among HCPs about the validity of the information the family provided [75]. Furthermore, HCPs experienced alerts and reminders to sometimes be burdensome, and they expressed concerns that symptom assessments were constant reminders of disease progression for the patients [51,52,60,62,70].

### Mutual Trust as a Prerequisite for Interpersonal Relationships

HCPs reported that telehealth provided reassurance, advice, a sense of control, and security, and it ensured the involvement of patients and families while improving the continuity of care [48-51,56,61,62,66,69,71,72,75]. HCPs felt more connected with patients and their families when using telehealth than through the telephone, as telehealth offered an opportunity for engagement and inquiry about the patients’ surroundings, making the consultations more personal [48,51,60,61]. Telehealth also facilitated 3-party consultations, which enabled the involvement of families and the creation of more trustworthy relationships [49,61,64]. It enabled guidance, the exchange of knowledge, education, and bilateral involvement between different HCPs working at different levels of health care, leading to feelings of improved collaboration, partnership, and peer support [49,58,66,67,72,73].

Nevertheless, HCPs did not always perceive telehealth to provide the same level of patient-tailored or in-depth care compared with in-person care [60,65,69,70]. In addition, HCPs expressed that it is challenging to discuss sensitive topics because they find it difficult to convey caring or empathy remotely [48,74]. These professionals also emphasized that telehealth requires a trusting relationship between different HCPs and between HCPs, patients, and their families [49,50,62,75]. They felt that video visits could be more appropriate for follow-up, as they experienced that previous in-person encounters with patients increased the comfort with and effectiveness of video consultations and helped form an overall picture of patients’ contexts and life situations [47,48,62].

## Discussion

### Principal Findings

This scoping review aimed to systematically map published studies that focus on HCPs’ experiences and perspectives on the use of telehealth in HBPC. Our findings suggest that telehealth is easy to use without being burdensome for HCPs, and it shows potential to improve time efficiency and the extension of palliative care services while enabling close connectedness with patients and families. Telehealth may also improve collaboration between HCPs working at different levels of health care, as well as make them more attentive and responsive to changes in patients’ symptoms or general well-being. However, problematic aspects of the use of telehealth in HBPC were also described, such as technical issues, resistance to change among HCPs, challenges with emotional support, ambiguous patient data, and the prerequisite of mutual trust and familiarity for the successful use of telehealth.

Our findings suggest that HCPs found telehealth easy to use for themselves and for patients, and they described positive outcomes for both patients and their families. Previous research on patients’ experiences of using telehealth supports the feasibility and relative ease of using telehealth among patients [14-17]. However, technological issues and legal concerns were reported in our review, which undermined HCPs’ confidence and made them revert to previous ways of working. HCPs faith in telehealth seems to be related to user-friendly, reliable, and personalized technology [33], and telehealth training should focus on maintaining HCPs’ confidence in providing care remotely [77]. Consistent with our findings, systematic reviews have found that HCPs have positive attitudes toward the use of technology but have expressed concerns regarding technical challenges and privacy [26,33]. HCPs who lack experience with the use of telehealth may have misconceptions regarding it, such as loss of personalized care, missing vital information, or assumptions that older patients would not be interested in or able to use telehealth [9,78]. This is supported by our findings showing that prior experience with the use of technology enhanced acceptance among HCPs.

Our findings indicate that telehealth adds value to HBPC by improving access to patients, time efficiency, quality, continuity, and coordination of care while increasing the capacity and extension of HBPC services. Similar descriptions have been found in previous systematic reviews [15-17]. Our review showed that telehealth provides an avenue for HCPs who rarely interact physically to come together over a digital medium. A lack of contact between different levels of health care may be a key barrier to successful collaboration [7], which is a challenge that could be met by telehealth [79]. However, our findings also showed that telehealth presented a new way of working, which was challenging to adjust to, and that a lack of motivation among HCPs reduced telehealth engagement. A mixed methods systematic review described that the use of telehealth requires substantial adjustment from the HCPs [77], and that resistance to change among HCPs is a barrier to the implementation of telehealth [80]. Although telehealth could reduce HCPs’ workload [27,34], HCPs may have concerns about telehealth increasing their workload [26,33] due to the required training in how to use it and the need for regular refresher courses [33,51,70]. If HCPs do not perceive telehealth to benefit workload or clinical practice, the solution will often not be adhered to or welcomed [81]. Researchers and policy makers should emphasize the potential benefits of using telehealth, rather than only focusing on how to operate it [81]. Our findings described that a lack of integration with existing health care services negatively affects the successful adoption of telehealth. Studies have described integration and interoperability issues as key aspects of negative user experiences [82,83]. HCPs’ motivations for any change depend on their ability to influence the change, be prepared for it, and value the change [84]. This underlines the importance of including HCPs when developing or implementing new technology for use in HBPC [85].

Our review describes telehealth as enabling HCPs to observe patients and their surroundings remotely, which was perceived as useful in clinical assessments and patient examinations. However, HCPs also expressed concerns regarding missing important patient information and the failure of video technology to convey important visual nuances. Research suggests that while video visits may offer a glimpse into patients’ lives and social contexts, they may not provide the same level of patient-tailored or in-depth care that in-person care provides [33]. Our findings indicate that patient-reported data were useful for supporting clinical assessments, improving symptom control, and helping HCPs prioritize patients. However, such data were sometimes perceived as confusing, creating uncertainty about what the proper actions would be. As the availability of patient-generated data increases, HCPs may want more uniformity in how to interpret patient-generated data and incorporate these into clinical decision-making [50].

A trusting relationship is a key factor in palliative care [13], and concerns have been raised whether the use of telehealth could affect the patient–HCP relationship and come at the expense of actual support and caregiving [13,22]. Our findings suggest that telehealth enabled meaningful and trusting relationships with patients and families and that it made HCPs more connected with patients and their families than through traditional follow-up. This is in line with an integrative review of the use of video in palliative care [33]. However, the goal of video consultations replacing a significant proportion of face-to-face care may be misplaced [80]. Based on our findings, we suggest that video consultations could be more appropriate for follow-up; HCPs emphasized that the usefulness of telehealth depends on all participants having existing trust and that previous in-person encounters with patients increased the comfort with and effectiveness of video consultations. Studies suggest that HCPs involved in palliative care may prefer the initial contact to be face-to-face [86], and HCPs may be skeptical of technologies that aim to replace all face-to-face encounters with patients [87].

Consistent with our review, a systematic review [88] found that HCPs may find it difficult to provide psychoemotional comfort and discuss end-of-life issues remotely [88]. Interestingly, this could be contrary to the perceptions of patients, as studies have shown that patients may find telehealth equal to or better than in-person consultations at providing emotional support, and they may consider it easier to discuss sensitive topics in the comfort of their own homes [14,89]. Understanding the potential of telehealth to support therapeutic relationships between patients and HCPs and being aware of the possible difficulties and tensions it may create are critical to its successful and acceptable use [13].

Our review included studies that were mainly conducted in high-income countries [90] in Europe and North America. Only 2 studies [52,67] were conducted in low- and middle-income countries (LMICs) [91]. The use of telehealth is growing in many LMICs [92]; however, the widespread adoption of telehealth in LMICs remains limited by resource scarcity, unreliable power, poor internet connectivity [93], and substantial infrastructure and regulatory barriers [92], which may explain why few studies conducted in LMICs were identified in our review.

There is an increasing trend to deliver palliative care services at home and to include diagnoses other than cancer [94]. However, in the majority of the studies included, telehealth was delivered to patients with cancer by specialized palliative care services located at hospitals. This may reinforce the impression that most palliative care services and research are still being conducted in cancer and hospital settings [95,96]. The studies in our review consisted of heterogeneous samples of HCPs, but nurses and physicians were the professionals included in most of the studies. Palliative care underlines the importance of an interdisciplinary team approach [97], and occupational therapists, psychologists, or social workers, for instance, could also play important roles when telehealth is used in HBPC [98]. Future studies need to address the experiences of using telehealth among more diverse HCPs working in settings other than hospitals with patients with diagnoses other than cancer. Finally, more research is needed in LMICs and in different cultural settings, as there may be other perspectives and experiences with the use of telehealth in HBPC across cultural settings.

### Limitations

Technology has developed rapidly over the last 2 decades, and some of the studies included in this scoping review describe the experiences of HCPs in using technology that is outdated compared with today’s standards. This may particularly be the case in terms of screen size, image resolution, color quality, and broadband issues, as mobile and network technology today offers significantly improved imaging technology and network stability compared with that 2 decades ago. However, our findings still highlight these important features from the point of view of HCPs, which will be vital to incorporate in future solutions. Despite our comprehensive and systematic search strategy, there may be studies that we have not been able to identify. Several terms are used for both telehealth and palliative care, and telehealth interventions for patients with incurable diseases or life-limiting illnesses may not have been classified as palliative care or telehealth intervention. Further, there exists a substantial amount of gray literature on this subject, which was not included since our review was limited to the inclusion of studies published in peer-reviewed journals. One of the studies [75] included described cultural barriers to the use of telehealth that were not described in the other studies. This suggests that there may be cultural barriers that we have not been able to fully identify and describe. Finally, our search strategy had language restrictions, as we included only studies in English, Nordic, Spanish, and Portuguese. However, as stated in the published protocol [40], we were able to include Chinese publications in the initial screening of published studies, although no relevant publications were identified. Due to these limitations, there may be experiences and perspectives from HCPs on the use of telehealth in HBPC we were not able to identify and describe.

### Conclusions

Overall, HCPs seem to find telehealth in HBPC easy to use without being burdensome. Our findings suggest that HCPs consider telehealth to improve patient outcomes in HBPC by providing patients and families with more personalized and accessible care. Telehealth enables HCPs to monitor patients more closely, and respond more quickly to changes in their symptoms or health status. Further, telehealth can help streamline processes, such as patient assessments or symptoms management, making it easier for HCPs to provide HBPC. Digital tools offered through telehealth can also facilitate improved communication between patients and HCPs, allowing for more convenient and effective care, while also enabling a close connectedness between HCPs, patients, and their families. Telehealth also seems to facilitate improved collaboration between professionals working at different levels of health care.

Despite these potential benefits, some HCPs may be hesitant to use telehealth in HBPC due to a lack of familiarity, being uncomfortable with the use of telehealth, or lacking the necessary training or resources to use it effectively. The use of telehealth in HBPC is a complex issue with both benefits and challenges, and opinions among HCPs will depend on a variety of factors, including their training, experience, and the specific technology being used. Substantial organizational hurdles need to be overcome in order to enable widespread adoption of telehealth in HBPC, and changes in practice and requirements for HCPs may overburden health care organizations that already lack the necessary workforce and resources. HCPs consider themselves to have central roles in implementing telehealth, and a lack of acceptance and motivation in this way of working is a key barrier to telehealth adoption. Policy makers and telehealth developers should be aware of this barrier when developing or implementing new technology for use in HBPC, highlighting the importance of user involvement.

## Appendix

Multimedia Appendix 1 PRISMA-ScR-Checklist.

Multimedia Appendix 2 Deviations from the published protocol.

Multimedia Appendix 3 Search strategy all databases.

Multimedia Appendix 4 Study characteristics.

## Abbreviations

HBPC: home-based palliative care
HCP: health care professional
LMIC: low- and middle-income country
PRISMA-ScR: PRISMA extension for scoping reviews

## Data Availability

The data collected, generated, and analyzed during the current study are publicly available as all the data were collected from published studies in peer-reviewed academic journals. An NVIVO file with the generated data sets is available from the corresponding author on reasonable request.
