# Supplementary material for: Health Care Professionals’ Experiences and Perspectives on Using Telehealth for Home-based Palliative Care: Scoping Review
Source: J Med Internet Res. 2023 Mar 29;25:e43429. doi: 10.2196/43429 (PMC10131609; doi:10.2196/43429)
Supplement: Multimedia Appendix 2 [file jmir_v25i1e43429_app2.pdf]

Multimedia appendix 2: Deviations from the published protocol

Planned approach described in the protocol	Type, description of the deviation from the protocol
**Inclusion and exclusion criteria:** We planned to include papers in English, Chinese, Portuguese, Spanish and Nordic languages based on authors' fluency	**Reduction:** We were able to screen Chinese publications in the initial title and abstract screening. However, the author from the published protocol that was fluent in Chinese was unable to participate in the published review. Thus, Chinese publications were not included. No relevant publications in Chinese were identified in the initial screening
**Data Collection Process:** We planned to use a standardized data charting form in the web application Covidence for data charting	**Change:** A data charting form was developed and used in the word processing software Microsoft Word, rather than in Covidence. This was for simplicity, flexibility and usability reasons. The data charting form was based on the standardized form in Covidence. Covidence was used to store and organize the studies included, as well as facilitate the screening and reviewing process
**Data Collection Process:** We planned to chart the following data from the included studies: authors, publication year, country, aim, sample, telehealth solution, design, and results	**Addition:** We decided to also chart how the included studies reported work experience among the included participants. This was done to provide a broader overview on participant characteristics, to hopefully guide or assist researchers that would want to determine whether a full systematic review is feasible
**Data Synthesis:** In the published protocol we planned that the first, second, third and last author would organize the data in the qualitative analysis software NVivo	**Reduction:** Based on a pragmatic choice, the first author organized the data and conducted the line-by-line coding in NVivo. The last author asked critical questions during each step of the analysis process to resolve differing interpretations during the coding process. All authors contributed with determining the final set of descriptive themes
