# Supplementary material for: Health Care Professionals’ Experiences and Perspectives on Using Telehealth for Home-based Palliative Care: Scoping Review
Source: J Med Internet Res. 2023 Mar 29;25:e43429. doi: 10.2196/43429 (PMC10131609; doi:10.2196/43429)
Supplement: Multimedia Appendix 3 [file jmir_v25i1e43429_app3.pdf]

Multimedia appendix 3: Search strategy all databases

DATABASE(S): OVID MEDLINE(R) ALL

SEARCH STRATEGY:

#	Searches
1	Palliative Care/ or "Hospice and Palliative Care Nursing"/ or exp Terminal Care/ or Palliative Medicine/ or exp Advance Care Planning/ or Terminally ill/
2	(palliative or palliate* or palliating).tw,kf.
3	((terminal* or "end stage*" or endstage* or "advanced stage*" or "late stage*") adj3 (disease* or ill* or care* or caring or treatment* or period* or nurs* or patient*)).tw,kf.
4	(eol or "end of life").tw,kf.
5	(advance*1 adj3 (plan*1 or planning or directive*)).tw,kf.
6	hospice*.tw,kf.
7	1 or 2 or 3 or 4 or 5 or 6
8	exp Telemedicine/
9	Telecommunications/ or exp Telemetry/ or Wireless Technology/ or exp Videoconferencing/
10	Mobile Applications/ or exp Telephone/ or computers/ or microcomputers/ or computers, handheld/ or smartphone/ or minicomputers/ or User-Computer Interface/ or Computer assisted instruction/
11	Internet-Based Intervention/
12	(((wearable or wireless) adj2 (technolog* or electronic*1 or device*1)) or (digital adj2 medicine) or (technolog* adj2 (remote or health)) or (remote adj2 care) or ((mobile or internet or electronic* or robot* or remote) adj2 (consultation* or application*1 or device*1))).tw,kf.
13	(telecommunicat* or tele-communicat* or teleconferenc* or tele-conferenc* or app or apps or app-based or mobile-based or "Short Message Service*" or sms or textmessag* or text-messag* or texting or videoconferenc* or video-conferenc* or webconferenc* or web-conferenc* or webcast* or web-cast* or webinar* or web-application* or web-based-application*).tw,kf.
14	(phone*1 or telephon* or smartphone* or smart-phone* or cellphone* or cell-phone* or mobilephone* or mobile-phone* or "personal digital assistant*" or palmpilot* or palm-pilot* or smarthome* or smart-home* or touchscreen* or "touch screen*" or "high tech*" or hightech*).tw,kf.
15	(telemedicin* or tele-medicin* or telehealth* or tele-health* or telecare* or tele-care* or telecari* or tele-cari* or emedic* or e-medic* or ehealth* or e-health* or mhealth* or m-health* or ehomecare* or e-homecare* or e-home-care* or telenurs* or tele-nurs* or teletherap* or tele-therap* or telerehab* or tele-rehab* or erehab* or e-rehab* or teleconsultation* or tele-consultation* or videoconsultation* or video-consultation* or telemonitor* or tele-monitor*).tw,kf.
16	("internet based intervention*" or "web based intervention*" or (digital adj2 intervention*)).tw,kf.

**17**	8 or 9 or 10 or 11 or 12 or 13 or 14 or 15 or 16
**18**	7 and 17
**19**	exp Independent Living/ or Homebound Persons/
**20**	exp Home Care Services/ or exp Community Health Nursing/
**21**	((assisted or independent) adj2 living).tw,kf.
**22**	"community dwelling".tw,kf.
**23**	(homecare* or home-care*).tw,kf.
**24**	(housebound or house-bound or homebound or home-bound or home or homes).tw,kf.
**25**	((community adj2 (healthcare* or health-care* or nurs*)) or (community adj2 health adj2 service*) or "district nurs*").tw,kf.
**26**	(remote or rural).tw,kf.
**27**	19 or 20 or 21 or 22 or 23 or 24 or 25 or 26
**28**	18 and 27
**29**	limit 28 to yr="2000 -Current"
**30**	limit 29 to (chinese or danish or english or norwegian or portuguese or spanish or swedish)

DATABASE(S): EMBASE

SEARCH STRATEGY:

**#**	**Searches**
**1**	exp palliative therapy/ or palliative nursing/ or exp terminal care/ or exp terminally ill patient/
**2**	(palliative or palliate* or palliating).tw,kw.
**3**	((terminal* or "end stage*" or endstage* or "advanced stage*" or "late stage*") adj3 (disease* or ill* or care* or caring or treatment* or period* or nurs* or patient*)).tw,kw.
**4**	(eol or "end of life").tw,kw.
**5**	(advance*1 adj3 (plan*1 or planning or directive*)).tw,kw.
**6**	hospice*.tw,kw.
**7**	1 or 2 or 3 or 4 or 5 or 6
**8**	exp telehealth/
**9**	exp telemetry/ or telecommunication/ or wireless communication/ or videoconferencing/
**10**	exp mobile application/ or telephone/ or mobile phone/ or smartphone/ or computer/ or microcomputer/ or personal computer/ or personal digital assistant/ or computer interface/ or human machine interface/
**11**	web-based intervention/
**12**	(((wearable or wireless) adj2 (technolog* or electronic*1 or device*1)) or (digital adj2 medicine) or (technolog* adj2 (remote or health)) or (remote adj2 care) or ((mobile or internet or electronic* or robot* or remote) adj2 (consultation* or application*1 or device*1))).tw,kw.

**13**	(telecommunicat* or tele-communicat* or teleconferenc* or tele-conferenc* or app or apps or app-based or mobile-based or "Short Message Service*" or sms or textmessag* or text-messag* or texting or videoconferenc* or video-conferenc* or webconferenc* or web-conferenc* or webcast* or web-cast* or webinar* or web-application* or web-based-application*).tw,kw.
**14**	(phone*1 or telephon* or smartphone* or smart-phone* or cellphone* or cell-phone* or mobilephone* or mobile-phone* or "personal digital assistant*" or palmpilot* or palm-pilot* or smarthome* or smart-home* or touchscreen* or "touch screen*" or "high tech*" or hightech*).tw,kw.
**15**	(telemedicin* or tele-medicin* or telehealth* or tele-health* or telecare* or tele-care* or telecari* or tele-cari* or emedic* or e-medic* or ehealth* or e-health* or mhealth* or m-health* or ehomecare* or e-homecare* or e-home-care* or telenurs* or tele-nurs* or teletherap* or tele-therap* or telerehab* or tele-rehab* or erehab* or e-rehab* or teleconsultation* or tele-consultation* or videoconsultation* or video-consultation* or telemonitor* or tele-monitor*).tw,kw.
**16**	("internet based intervention*" or "web based intervention*" or (digital adj2 intervention*)).tw,kw.
**17**	8 or 9 or 10 or 11 or 12 or 13 or 14 or 15 or 16
**18**	7 and 17
**19**	independent living/ or homebound patient/
**20**	exp home care/ or exp community health nursing/
**21**	((assisted or independent) adj2 living).tw,kw.
**22**	"community dwelling".tw,kw.
**23**	(homecare* or home-care*).tw,kw.
**24**	(housebound or house-bound or homebound or home-bound or home or homes).tw,kw.
**25**	((community adj2 (healthcare* or health-care* or nurs*)) or (community adj2 health adj2 service*) or "district nurs*").tw,kw.
**26**	(remote or rural).tw,kw.
**27**	19 or 20 or 21 or 22 or 23 or 24 or 25 or 26
**28**	18 and 27
**29**	limit 28 to yr="2000 -Current"
**30**	limit 29 to (chinese or danish or english or norwegian or portuguese or spanish or swedish)

DATABASE(S): APA PSYCINFO

SEARCH STRATEGY:

#	Searches
**1**	palliative care/ or hospice/ or advance directives/ or terminally ill patients/
**2**	(palliative or palliate* or palliating).tw.

**3**	((terminal* or "end stage*" or endstage* or "advanced stage*" or "late stage*") adj3 (disease* or ill* or care* or caring or treatment* or period* or nurs* or patient*)).tw.
**4**	(eol or "end of life").tw.
**5**	(advance*1 adj3 (plan*1 or planning or directive*)).tw.
**6**	hospice*.tw.
**7**	1 or 2 or 3 or 4 or 5 or 6
**8**	exp electronic health services/
**9**	digital technology/ or exp artificial intelligence/ or exp computer applications/ or exp electronic communication/ or exp mobile technology/ or exp streaming technology/ or exp wireless technologies/
**10**	computers/ or exp microcomputers/ or exp mobile devices/
**11**	(((wearable or wireless) adj2 (technolog* or electronic*1 or device*1)) or (digital adj2 medicine) or (technolog* adj2 (remote or health)) or (remote adj2 care) or ((mobile or internet or electronic* or robot* or remote) adj2 (consultation* or application*1 or device*1))).tw.
**12**	(telecommunicat* or tele-communicat* or teleconferenc* or tele-conferenc* or app or apps or app-based or mobile-based or "Short Message Service*" or sms or textmessag* or text-messag* or texting or videoconferenc* or video-conferenc* or webconferenc* or web-conferenc* or webcast* or web-cast* or webinar* or web-application* or web-based-application*).tw.
**13**	(phone*1 or telephon* or smartphone* or smart-phone* or cellphone* or cell-phone* or mobilephone* or mobile-phone* or "personal digital assistant*" or palmpilot* or palm-pilot* or smarthome* or smart-home* or touchscreen* or "touch screen*" or "high tech*" or hightech*).tw.
**14**	(telemedicin* or tele-medicin* or telehealth* or tele-health* or telecare* or tele-care* or telecari* or tele-cari* or emedic* or e-medic* or ehealth* or e-health* or mhealth* or m-health* or ehomecare* or e-homecare* or e-home-care* or telenurs* or tele-nurs* or teletherap* or tele-therap* or telerehab* or tele-rehab* or erehab* or e-rehab* or teleconsultation* or tele-consultation* or videoconsultation* or video-consultation* or telemonitor* or tele-monitor*).tw.
**15**	("internet based intervention*" or "web based intervention*" or (digital adj2 intervention*)).tw.
**16**	8 or 9 or 10 or 11 or 12 or 13 or 15
**17**	7 and 16
**18**	self-care skills/
**19**	home care/
**20**	homebound/
**21**	((assisted or independent) adj2 living).tw.
**22**	"community dwelling".tw.

**23**	(homecare* or home-care*).tw.
**24**	(housebound or house-bound or homebound or home-bound or home or homes).tw.
**25**	((community adj2 (healthcare* or health-care* or nurs*)) or (community adj2 health adj2 service*) or "district nurs*").tw.
**26**	(remote or rural).tw.
**27**	18 or 19 or 20 or 21 or 22 or 23 or 24 or 25 or 26
**28**	17 and 27
**29**	limit 28 to yr="2000 -Current"
**30**	limit 29 to (chinese or danish or english or norwegian or portuguese or spanish or swedish)

DATABASE(S): AMED (ALLIED AND COMPLEMENTARY MEDICINE)

**SEARCH STRATEGY:**	
**#**	**Searches**
**1**	palliative care/ or exp terminal care/ or terminal illness/ or advance directives/
**2**	(palliative or palliate* or palliating).tw.
**3**	((terminal* or "end stage*" or endstage* or "advanced stage*" or "late stage*") adj3 (disease* or ill* or care* or caring or treatment* or period* or nurs* or patient*)).tw.
**4**	(eol or "end of life").tw.
**5**	(advance*1 adj3 (plan*1 or planning or directive*)).tw.
**6**	hospice*.tw.
**7**	1 or 2 or 3 or 4 or 5 or 6
**8**	telemedicine/
**9**	telecommunications/ or telephone/
**10**	exp computers/ or exp medical informatics/ or computer assisted instruction/
**11**	(((wearable or wireless) adj2 (technolog* or electronic*1 or device*1)) or (digital adj2 medicine) or (technolog* adj2 (remote or health)) or (remote adj2 care) or ((mobile or internet or electronic* or robot* or remote) adj2 (consultation* or application*1 or device*1))).tw.
**12**	(telecommunicat* or tele-communicat* or teleconferenc* or tele-conferenc* or app or apps or app-based or mobile-based or "Short Message Service*" or sms or textmessag* or text-messag* or texting or videoconferenc* or video-conferenc* or webconferenc* or web-conferenc* or webcast* or web-cast* or webinar* or web-application* or web-based-application*).tw.

**13**	(phone*1 or telephon* or smartphone* or smart-phone* or cellphone* or cell-phone* or mobilephone* or mobile-phone* or "personal digital assistant*" or palmpilot* or palm-pilot* or smarthome* or smart-home* or touchscreen* or "touch screen*" or "high tech*" or hightech*).tw.
**14**	(telemedicin* or tele-medicin* or telehealth* or tele-health* or telecare* or tele-care* or telecari* or tele-cari* or emedic* or e-medic* or ehealth* or e-health* or mhealth* or m-health* or ehomecare* or e-homecare* or e-home-care* or telenurs* or tele-nurs* or teletherap* or tele-therap* or telerehab* or tele-rehab* or erehab* or e-rehab* or teleconsultation* or tele-consultation* or videoconsultation* or video-consultation* or telemonitor* or tele-monitor*).tw.
**15**	("internet based intervention*" or "web based intervention*" or (digital adj2 intervention*)).tw.
**16**	8 or 9 or 10 or 11 or 12 or 13 or 14 or 15
**17**	7 and 16
**18**	exp independent living/
**19**	community health nursing/ or exp home care services/
**20**	((assisted or independent) adj2 living).tw.
**21**	"community dwelling".tw.
**22**	(homecare* or home-care*).tw.
**23**	(housebound or house-bound or homebound or home-bound or home or homes).tw.
**24**	((community adj2 (healthcare* or health-care* or nurs*)) or (community adj2 health adj2 service*) or "district nurs*").tw.
**25**	(remote or rural).tw.
**26**	18 or 19 or 20 or 21 or 22 or 23 or 24 or 25
**27**	17 and 26
**28**	limit 27 to yr="2000 -Current"
**29**	limit 28 to (chinese or danish or english or norwegian or portuguese or spanish or swedish)

DATABASE(S): CINAHL COMPLETE

SEARCH STRATEGY:

**#**	**Query**
**S30**	S18 AND S27
**S29**	S18 AND S27

**S28**	S18 AND S27
**S27**	S19 OR S20 OR S21 OR S22 OR S23 OR S24 OR S25 OR S26 OR S26
**S26**	TI (remote or rural) or AB (remote or rural)
**S25**	TI ((community N1 (healthcare* or health-care* or nurs*)) or (community N1 health N1 service*) or "district nurs*") or AB ((community N1 (healthcare* or health-care* or nurs*)) or (community N1 health N1 service*) or "district nurs*")
**S24**	TI (housebound or house-bound or homebound or home-bound or home or homes) or AB (housebound or house-bound or homebound or home-bound or home or homes)
**S23**	TI (homecare* or home-care*) or AB (homecare* or home-care*)
**S22**	TI "community dwelling" or AB "community dwelling"
**S21**	TI ((assisted or independent) N1 living) OR AB ((assisted or independent) N1 living)
**S20**	(MH "Home Health Care+") OR (MH "Home Nursing") OR (MH "Community Health Nursing+")
**S19**	(MH "Community Living+") OR (MH "Homebound Persons")
**S18**	S7 AND S17
**S17**	S8 OR S9 OR S10 OR S11 OR S12 OR S13 OR S14 OR S15 OR S16
**S16**	TI ("internet based intervention*" or "web based intervention*" or (digital N1 intervention*)) or AB ("internet based intervention*" or "web based intervention*" or (digital N1 intervention*))
**S15**	TI (telemedicin* or tele-medicin* or telehealth* or tele-health* or telecare* or tele-care* or telecari* or tele-cari* or emedic* or e-medic* or ehealth* or e-health* or mhealth* or m-health* or ehomecare* or e-homecare* or e-home-care* or telenurs* or tele-nurs* or teletherap* or tele-therap* or telerehab* or tele-rehab* or erehab* or e-rehab* or teleconsultation* or tele-consultation* or videoconsultation* or video-consultation* or telemonitor* or tele-monitor*) OR AB (telemedicin* or tele-medicin* or telehealth* or tele-health* or telecare* or tele-care* or telecari* or tele-cari* or emedic* or e-medic* or ehealth* or e-health* or mhealth* or m-health* or ehomecare* or e-homecare* or e-home-care* or telenurs* or tele-nurs* or teletherap* or tele-therap* or telerehab* or tele-rehab* or erehab* or e-rehab* or teleconsultation* or tele-consultation* or videoconsultation* or video-consultation* or telemonitor* or tele-monitor*)
**S14**	TI (phone or phones or telephon* or smartphone* or smart-phone* or cellphone* or cell-phone* or mobilephone* or mobile-phone* or "personal digital assistant*" or palmpilot* or palm-pilot* or smarthome* or smart-home* or touchscreen* or "touch screen*" or "high tech*" or hightech*) or AB (phone or phones or telephon* or smartphone* or smart-phone* or cellphone* or cell-phone* or mobilephone* or mobile-phone* or "personal digital assistant*" or palmpilot* or palm-pilot* or smarthome* or smart-home* or touchscreen* or "touch screen*" or "high tech*" or hightech*)

**S13**	TI (telecommunicat* or tele-communicat* or teleconferenc* or tele-conferenc* or app or apps or app-based or mobile-based or "Short Message Service*" or sms or textmessag* or text-messag* or texting or videoconferenc* or video-conferenc* or webconferenc* or web-conferenc* or webcast* or web-cast* or webinar* or web-application* or web-based-application*) OR AB (telecommunicat* or tele-communicat* or teleconferenc* or tele-conferenc* or app or apps or app-based or mobile-based or "Short Message Service*" or sms or textmessag* or text-messag* or texting or videoconferenc* or video-conferenc* or webconferenc* or web-conferenc* or webcast* or web-cast* or webinar* or web-application* or web-based-application*)
**S12**	TI (((wearable or wireless) N1 (technolog* or electronic or electronics or device or devices)) or (digital N1 medicine) or (technolog* N1 (remote or health)) or (remote N1 care) or ((mobile or internet or electronic* or robot* or remote) N1 (consultation* or application or applications or device or devices))) or AB (((wearable or wireless) N1 (technolog* or electronic or electronics or device or devices)) or (digital N1 medicine) or (technolog* N1 (remote or health)) or (remote N1 care) or ((mobile or internet or electronic* or robot* or remote) N1 (consultation* or application or applications or device or devices)))
**S11**	(MH "Internet-Based Intervention")
**S10**	(MH "Mobile Applications") OR (MH "Telephone+") OR (MH "Microcomputers+") or (MH "Computer Assisted Instruction")
**S9**	(MH "Telemetry") OR (MH "Telecommunications") OR (MH "Videoconferencing+") OR (MH "Wireless Communications") OR (MH "Teleconferencing")
**S8**	(MH "Telehealth+")
**S7**	S1 OR S2 OR S3 OR S4 OR S5 OR S6
**S6**	TI hospice* or AB hospice*
**S5**	TI (advance* N2 (plan or plans or planning or directive*)) or AB (advance* N2 (plan or plans or planning or directive*))
**S4**	TI (eol or "end of life") or AB (eol or "end of life")
**S3**	TI ((terminal* or "end stage*" or endstage* or "advanced stage*" or "late stage*") N2 (disease* or ill* or care* or caring or treatment* or period* or nurs* or patient*)) or AB ((terminal* or "end stage*" or endstage* or "advanced stage*" or "late stage*") N2 (disease* or ill* or care* or caring or treatment* or period* or nurs* or patient*))
**S2**	TI (palliative or palliate* or palliating) or AB (palliative or palliate* or palliating)
**S1**	(MH "Hospice and Palliative Nursing") OR (MH "Palliative Medicine") OR (MH "Palliative Care") OR (MH "Terminal Care+") OR (MH "Terminally Ill Patients+") OR (MH "Advance Care Planning")

SEARCH HISTORY WEB OF SCIENCE

**INDEXES=SCI-EXPANDED, SSCI, A&HCI, ESCI**		
Set		
**# 23**	(#20 and #13) *AND* LANGUAGE: (English OR Chinese OR Danish OR Norwegian OR Portuguese OR Spanish OR Swedish)	*Timespan= 2000-2021*
# 22	#20 and #13	*Timespan= 2000-2021*
**# 21**	#20 AND #13	
**# 20**	#19 OR #18 OR #17 OR #16 OR #15 OR #14	
# 19	TS=(remote or rural)	
**# 18**	TS=((community NEAR/1 (healthcare* or health-care* or nurs*)) or (community NEAR/1 health NEAR/1 service*) or "district nurs*")	
**# 17**	TS=(housebound or house-bound or homebound or home-bound or home or homes)	
# 16	TS=(homecare* or home-care*)	
# 15	TS="community dwelling"	
**# 14**	TS=((assisted or independent) NEAR/1 living)	
# 13	#12 AND #6	
# 12	#11 OR #10 OR #9 OR #8 OR #7	
**# 11**	TS=("internet based intervention*" or "web based intervention*" or (digital NEAR/1 intervention*))	
**# 10**	TS=(telemedicin* or tele-medicin* or telehealth* or tele-health* or telecare* or tele-care* or telecari* or tele-cari* or emedic* or e-medic* or ehealth* or e-health* or mhealth* or m-health* or ehomecare* or e-homecare* or e-home-care* or telenurs* or tele-nurs* or teletherap* or tele-therap* or telerehab* or tele-rehab* or erehab* or e-rehab* or teleconsultation* or tele-consultation* or videoconsultation* or video-consultation* or telemonitor* or tele-monitor*)	

# 9	TS=(phone or phones or telephon* or smartphone* or smart-phone* or cellphone* or cell-phone* or mobilephone* or mobile-phone* or "personal digital assistant*" or palmpilot* or palm-pilot* or smarthome* or smart-home* or touchscreen* or "touch screen*" or "high tech*" or high tech*)
**# 8**	TS=(telecommunicat* or tele-communicat* or teleconferenc* or tele-conferenc* or app or apps or app-based or mobile-based or "Short Message Service*" or sms or textmessag* or text-messag* or texting or videoconferenc* or video-conferenc* or webconferenc* or web-conferenc* or webcast* or web-cast* or webinar* or web-application* or web-based-application*)
# 7	TS= (((wearable or wireless) NEAR/1 (technolog* or electronic or electronics or device or devices)) or (digital NEAR/1 medicine) or (technolog* NEAR/1 (remote or health)) or (remote NEAR/1 care) or ((mobile or internet or electronic* or robot* or remote) NEAR/1 (consultation* or application or applications or device or devices)))
**# 6**	#5 OR #4 OR #3 OR #2 OR #1
**# 5**	TS = hospice*
# 4	TS =(advance* NEAR/2 (plan or plans or planning or directive*))
**# 3**	TS=(eol or "end of life")
**# 2**	TS=((terminal* or "end stage*" or endstage* or "advanced stage*" or "late stage*") NEAR/2 (disease* or ill* or care* or caring or treatment* or period* or nurs* or patient*))
**# 1**	TS= (palliative or palliate* or palliating)
