# Supplementary material for: Health Care Professionals’ Experiences and Perspectives on Using Telehealth for Home-based Palliative Care: Scoping Review
Source: J Med Internet Res. 2023 Mar 29;25:e43429. doi: 10.2196/43429 (PMC10131609; doi:10.2196/43429)
Supplement: Multimedia Appendix 4 [file jmir_v25i1e43429_app4.pdf]

Multimedia Appendix 4. Characteristics of the included studies.

Author, year, Country	Aim	Sample	Telehealth application	Design and methods	Results
Adam et al., 2020 UK	To develop a novel digital intervention to optimize cancer pain control in the community and describe the intervention development, content and rationale, and the initial feasibility	4 HCPs[1] (2 nurses and 2 general practitioner); sex NR[2], age NR, experience NR	"Can-Pain" app for pain monitoring and management	Qualitative; interview study. Intervention mapping approach	Can-Pain highlighted unrecognized problems, promoted shared understanding about symptoms between patients and HCPs and supported shared decision-making
Alodhayani et al., 2021 Saudi Arabia	To explore the specific cultural factor related to patients and their caregivers from the perspective of physicians, nurses, and trainers that have influenced the pilot implementation of remotely accessible healthcare at home	7 hospital-based HCPs (4 nurses, 2 family physicians, 1 technologist); 4 males, age NR, experience NR	Various digital health-tools such as mobile apps remote monitoring devices (glucometers, blood pressure monitors, oximeters, thermometers), teleconsultation, e-response centers	Phenomenological qualitative study; focus groups interview. Framework analysis	Telehealth enabled HCPs to provide guidance and training to the families in the use of medical devices remotely. HCPs experienced discrepancy in information shared by patients and caregivers and that caregivers remotely could exaggerate the severity of the situation. Some HCPs experienced that families could be reluctant to film the patient, making assessments difficult. In Saudi Arabian culture, women may be reluctant to show their face on camera. HCPs were reluctant in requesting that female patients expose body parts on camera for clinical assessments. HCPs expressed that patients seemed to not take video-consultations as seriously. Relying on family caregivers to facilitate video-consultations was also a limiting factor, as many family caregivers were unavailable at the time of consults
Bhargava et al., 2021 Canada	To demonstrate RELIEF to be a feasible tool for patients with PC[3] needs to easily self-report their symptoms to their clinical team; and to allow for timely interventions or close monitoring by clinical staff to minimize unnecessary visits to the emergency department or admissions to hospital in this patient population	PC nurses and physicians (n= NR), sex NR, age NR, experience NR	RELIEF web app for patient-reported symptoms. Symptom-reporting generated alerts to specialized PC teams	Survey data from the PC nurses and physicians who participated in the pilot	The majority of HCPs reported improved confidence in providing care and an improved client experience quality of life. There were also four calls made by patients and clinical staff for technical support. The patient-generated data made HCPs more responsive to changes in patients' conditions, and made it possible to manage patients in the home, and prevent hospital admissions
Bonsignore et al., 2018 USA	To describe a telehealth PC program using remote patient monitoring application and videoconferencing; evaluate the feasibility, usability, and	2 HCPs (caregivers) and 2 telehealth providers, sex NR, age NR , experience NR	App on tablet that facilitated communication between patient/caregiver and HCPs, app to report	Mixed-methods evaluation approach;	HCPs reported a greater ability to expand their caseload due to the facilitation of direct, efficient contact with patients, including patients in rural areas. The solution improved efficiency of medication refills, easier symptom checks, and increased comfort and peace of mind. HCPs described abilities

Author, year, Country	Aim	Sample	Telehealth application	Design and methods	Results
	acceptability; and use a quality data assessment collection tool in addition to digital ratings of symptom burden and hospice transitions		symptoms, management of medication, send messages, and for videoconferencing	HCPs participated in semi-structured interviews	to comfort patients' emotional and spiritual needs through digital messages. Challenges were that the system could not replace the depth of in-person care
Cameron, 2021 USA	To examine the comfort of hospice staff using telehospice to connect virtually with patients and caregivers.	44 HCPs (1 physician, 25 nurses, 2 nurse practitioners, 12 social workers, 3 spiritual care providers, 2 administrators); age range 27-69, 42 females, bachelor's degree (n=16), master's, (n = 12, 27.9%) and associates degree (n=12)	Alpha Virtual Assist (AVA) a remote patient monitoring and a communication platform	Mixed-methods; quantitative and qualitative survey questionnaire	Most participants were comfortable using telehealth, and were able to adjust it to different settings. HCPs emphasized the benefit of being able to visualize the patient, and improved involvement of the families. Telehealth enabled improved family support and comfort. HCPs experienced that patients felt more secure with easy access to HCPs. The visual features enabled HCPs to notice signs of deterioration, and could give patients advice on how to use medical equipment such as inhalers. Telehealth enabled improved clinical assessments. Connectivity issues were reported. HCPs wished they received more training, and that a chatting feature was available. No difference in comfort between nurses and other HCPs
Collier et al. 2016 Australia	To explore clinicians' perspectives on and experiences of the utilization of a pilot telehealth model and its integration into a specialist community palliative care program	10 HCPs, (6 community specialist nurses, 2 nurse practitioners, 1 family carer liaison nurse, 1 NR), 1 male, 9 females, age NR, experience NR	Self-report assessment tools for patients and carers and remote activity monitoring; ongoing video-based conferences between service staff, patient or carer; and virtual case conferences with patient and carer, service staff and general practitioners	Qualitative; focus group interviews	Telehealth was considered timesaving and ideal to support rural and remote patients, and provided opportunity for working smarter and more efficiently and safely. The telehealth solution made clinical assessments easier, and provided nurses with meaningful and timely, patient-reported, clinical data. Challenges were technological instability, lack of physical assessments, patients manipulating the screening tool to initiate or avoid triggers and responding to burdensome alerts
Eastman et al. 2021 Australia	To understand patient and HCPs perspectives on the use of telehealth within community PC practice	22 HCPs responded with 18 involved in telehealth consultations; sex NR, age NR, experience not reported	Video consultations for follow-up	Quantitative; survey	HCPs had concerns with video conferencing in relation to patient engagement with new technology and technological problems. Most HCPs preferred video conferencing to telephone calls and all preferred face to face interactions to telehealth. 89% indicated a preference for ongoing service provision combing face to face and telehealth consultations

Author, year, Country	Aim	Sample	Telehealth application	Design and methods	Results
Funderskov et al., 2019 Denmark	To explore the advantages and disadvantages of using video consultations, as experienced by SPC[4] healthcare professionals, who are involved in PC at home	8 HCP (5 community nurses, 1 physician, 1 physiotherapist); sex NR, age NR , experience NR	Tablets for video consultations	Qualitative study; hermeneutic post phenomenolog ical approach	The use of tablets in video consultations facilitated direct palliative care and cooperation between community nurses and the SPC team. Video consultations mediated active patient and relative involvement, access to care, room for co-operation for health-care professionals and the use of a technical device in SPC. Potential barriers against using video consultations are the discussions about personal and private issues regarding the illness, while family members are present
Hackett et al., 2020 UK	To explore HCPs and patients' perceptions of their engagement with an information and communication technology system for pain management to understand the mechanisms that could support implementation into routine PC practice	12 clinical nurse specialists, sex NR, age NR, experience NR	Information and communication technology system for pain management (assessment and monitoring) called PainCheck. And a self-management educational booklet and video called Tackling cancer pain	Qualitative study; semi-structured interviews, thematic analysis	The role of health professionals was a key component to patient engagement with the information and communication technology system. Where patients engaged with the information and communication technology system, both patients and health professionals reported benefits to system use in addition to usual care
Harding et al., 2021 India, Uganda, Zimbabwe	To design a mobile phone application to enable or improve communication between family caregivers, community caregivers, and PC teams; to evaluate its acceptability, processes, and mechanisms of action; and to propose refinements	9 HCPs (among 4 nurses, 2 doctors); 2 males, age NR, experience NR	App to communicate patient-reported outcomes to their PC providers each week on a data dashboard	Co-design for app development; Qualitative data on the user experience	The app provided better understanding of patients' symptoms, concerns, and outcomes of care in "real time" with regular ongoing assessment which enabled individualized care. The app was user-friendly and enabled integrated patient-reported outcome assessment. This enabled HCPs to prioritize patients. Disadvantages with the app were: reliance on internet connectivity; disappointment by patients who expected to receive treatment immediately; requirement that the HCPs be literate; lack of confirmation for HCPs that their upload was received. A key challenge was the ability to learn everything needed, although over time users provided peer support to use the app efficiently
Haydon et al., 2021 Australia	To explore the costs, service activity and staff experiences resulting from the introduction of telehealth in a community PC service	15 HCPs (6 nursing, 5 allied health, 2 medical, 2 administrative); 12 females, 3 males, median age 48 (range	Videoconferencing between patients and HCPs working at the PC unit at the hospital	Qualitative; semi-structured interviews with HCPs. Quantitative	HCPs felt that telehealth enabled more patient-centered care and improved efficiency, better involvement of families, more responsive to patients' needs, reduced travel and increased access to care for patients. Telehealth increased peer support through more involvement between organizations, which

Author, year, Country	Aim	Sample	Telehealth application	Design and methods	Results
		30-65) years, experience NR		data for cost and service activity data	increased professional development as knowledge was easier shared
Helleman et al., 2020 The Netherlands	To evaluate the use of ALS[5] Home-monitoring and Coaching in specialist ALS care, and the user experiences from the perspectives of patients and HCPs	9 HCPs (2 rehabilitation physicians, 2 occupational therapists, 2 physical therapists, 1 speech therapist, 1 dietician and 1 social worker); sex NR, age NR, experience NR	ALS-app for self-monitoring and messaging, alerts for symptom-worsening, and nurse practitioner follow-up. Patients self-monitored their well-being (daily report), body weight (weekly) and functional status (monthly)	Qualitative; survey of HCPs	The majority of HCPs used the monitored data to prepare for consultations. Workload was similar compared to care without telehealth, but in-person consultations were used more effectively. The available monitoring data helped them prepare the consultation. All HCPs reported that the use of telehealth added value to the ALS care and would recommend it to others
Hochstenbach et al., 2016 The Netherlands	To explore feasibility of the mobile application for patients, the web application for nurses, and the integration of both applications in routine clinical practice	3 registered nurses specialized in pain and palliative care; 1 male, mean age 52 (SD 2, range 50-54) years, 10-15 years of experience of working in PC consultation team	Mobile app for patients that was connected to a web application for nurses. Patient's situation were monitored and analysed (completed pain diaries, scheduled and actual medication intake and text messages) once every workday. nurses consult patients by text messages and phone	Qualitative	The app provided nurses with risk flags while how to act was up to the nurses. Nurses responded differently to patients and situations. Nurses preferred more uniformity for the sake of clinical and research practice. They were unsure whether the patients became more or less independent using the app. Collaboration with the treating physician, general practitioner and pharmacist was crucial. Nurses were enthusiastic about the app but needed time to get used to this way of working. They trusted the intervention. Nurses suggested that questions contained more information about the cause of their symptoms, and that patients were asked if they wanted the nurse to contact you based on the diary answers
Lind et al., 2007 Sweden	To explore and describe professional caregivers' experiences of palliative home health-care patients' use of pain diaries and digital pen technology for frequent pain assessment	5 HCP (3 nurses, 2 physicians and 1 secretary); 1 male, age range 37-62 years, experience range 1½-15 years	Paper pain diary (pain intensity, number of consumed extra doses of analgesics) and digital pen technology. Checked and printed by secretary or nurse responsible	Qualitative descriptive and explorative	HCP showed a shifting outlook towards the pain-assessment method, an initial cautious outlook due to low expectations of the patients' abilities to use the pain assessment method. The HCP experienced positive outcomes in terms of an increased awareness of pain, and positive patient influences including increased participation in their care, increased security, and improved changes in pain treatment as a response to reported pain assessments
McCall et al., 2008	Preliminary study to test the acceptability and usability of the	9 HCPs completed the first questionnaire, 8	A mobile phone-based technology (ASyMSp) to	Mixed methods; pre-	The post-study questionnaires and interviews revealed that most of the HCPs' were positive about the use of ASyMSp in

Author, year, Country	Aim	Sample	Telehealth application	Design and methods	Results
UK	Advanced Symptom Management system in palliative care (ASyMSp), a developed version of the symptom assessment software (ASyMS)	HCP completed the second questionnaire, 4 HCP were interviewed, profession, sex NR, age NR, experience NR	monitor and manage symptoms reported by patients cared for at home in the advanced stages of illness	and post-study surveys, semi-structured interviews	monitoring, management and assessment of patient's symptoms. The HCPs' gave mixed comments about the range of symptoms pre-defined in the ASyMSp system. Most HCPs considered the ASyMSp system to be acceptable for the early stage of the study, but needed to be expanded and personalized in future studies. HCPs were uncertain whether the ASyMSp system saved time and resources
Miyazaki et al., 2003 Canada	To examine the usability of an ISDN-based videophone for home care	15 HCPs (4 case managers, 1 resource coordinator, 1 respiratory therapist, 8 nurses (including casual staff)); 1 male; age NR , experience NR	ISDN videophone, HCPs received training in groups on how to operate videophone	Multi-method	Many HCPs believed that the effect of the videophone on care depended on the needs of individual patient. HCPs enjoyed seeing the image of the patients because they could tell how a patient was feeling by looking for physical symptoms as well as cues to the patients' emotional state.<br>HCP believed that the videophone was instrumental in educating other professionals and in helping a patient during an emergency
Nguyen et al., 2020 USA	To describe the challenges we encountered in conducting the HomePal study during 'normal' times and important lessons learnt and recommendations for the field, anchored in two common technology adoption and implementation frameworks to help inform future approaches to implementing and evaluating similar interventions within complex adaptive systems	111 nurses, sex NR, age NR, experience NR	HomePal, just briefly described in the current paper. Synchronous video consultation with a remote physician while a nurse was in the patient's home	Pragmatic, cluster randomized non-inferiority trial across 14 sites	A low uptake of the video-intervention. The authors have addressed the experiences with the video-intervention according to NASSS[6] framework which gives rich data on the experiences with the intervention and why they had to prematurely stop the trial after 12 months. The HCP interviews revealed challenges with: healthcare organizations' readiness for technology; the required change of practice; the clinical complexity in HBPC[7]; communication issues, decision-making and care delivery challenges; concerns over quality of care as well as technological issues.
Oelschlägel et al., 2021 Norway	To explore municipal HCPs' experiences regarding the significant challenges, facilitators, and assessments associated with implementing a technological solution named "remote home care" in palliative home care for patients with cancer	8 HCPs (2 specialized nurses, 2 nurses, 1 social worker, 1 physical therapist, 2 occupational therapists), 6 female, 2 male. Year experience from health care mean (range): 13 (4–27),	A telehealth app "RHC"[8], enables HCP to remotely monitor and manage patients' safety, security, wellness, treatment, and care	Qualitative; focus-group interviews and individual interviews	HCPs perceived that RHC contributed to improvements in the coordination and continuity of care, and enhanced patients' feelings of safety which was considered beneficial for both patients and their families. HCPs expressed worries regarding missing important patient information. The lack of integration across health-care services and the unfamiliarity of RHC were explained as representing a shift in responsibility from the health-care system to patients. HCPs found it challenging to assess and understand the patients' care needs based only on

Author, year, Country	Aim	Sample	Telehealth application	Design and methods	Results
		years experience from current position: 6 (1-10).			remote measures of vital signs. The availability to discuss difficult issues was highlighted as being a fundamental resource for facilitating the provision of care
Osuji et al., 2020 USA	To assess the usefulness and appropriateness (i.e., value proposition) of video visits from the perspective of HBPC physicians and nurses	84 HCPs (36 physicians, 48 registered nurse), sex NR, age NR, experience NR	Video visits in HBPC	Cross-sectional anonymous survey; quantitative and qualitative approach	Participants had favorable attitudes toward video visits and telehealth. Respondents felt confident in the skills needed to make their role in video visits successful. Clinicians were neutral on whether video visits were useful for their practice or enhanced the patient–caregiver experience. Clinicians found video visits to be most appropriate for follow-up care (as opposed to start of care). The interviews yielded two themes that complemented the survey findings: (1) factors enhancing the value proposition (positive responses from patients and families and convenience) and (2) factors diminishing the value proposition (issues related to the technology and scheduling)
Read Paul et al., 2019 Canada	To gain a preliminary understanding of the experience of using mobile web-based videoconferencing (WBVC) for conducting in-home PC care consults with elderly rural patients with life-limiting illness	13 HCPs (9 home care nurses; 2 clinical nurse specialists; 2 physician consultants. Sex NR, age NR, experience NR	Laptop computer with webcam and speakerphone to connect to a distant PC physician consultant	Descriptive, exploratory, proof-of-concept study	Participants reported they were comfortable discussing concerns by WBVC and felt it was an acceptable and convenient way to address needs. Audiovisual quality was not ideal but was adequate for communication. Use of WBVC improved access and saved time and travel. Fears were expressed about lack of security of information transmitted over the Internet
Scofano et al., 2022 Brazil	To evaluate whether telemedicine provided through telemonitoring can improve the ongoing relationship between the doctor, the nurse and the patient	12 nurses; 24% males, mean age 43 (SD 5) years, year experience mean: 5,5	Telemonitoring during home dialysis for patients diagnosed with end-stage renal disease	Mixed methods. Qualitative semi-structured interviews with nurses, quantitative questionnaires	Initially nurses lacked confidence; however, with experience they became more comfortable with telehealth and were able to use it with ease. Telehealth helped with decision-making, and HCPs did not feel that telehealth compromised privacy. Nurses experienced improved interprofessional collaboration and support from the treating physician
Shulver et al., 2016 Australia	To examine healthcare worker views on telehealth, and their implications for implementation to mainstream healthcare services for older people	44 healthcare workers within rehabilitation, allied health, residential care or PC,	Most frequently videoconferencing, iPad, and monitoring over a distance.	Qualitative, focus groups, thematic analysis	The views of participants varied with the extent of telehealth experience and perception of accessibility of healthcare services. Four themes describing clinician attitudes and perceptions that could impact implementation of telehealth services were: Workability of telehealth: exponential growth

Author, year, Country	Aim	Sample	Telehealth application	Design and methods	Results
		sex NR, age NR, experience NR			in access or decay in the quality of healthcare? What is an acceptable level of risk to patient safety with telehealth? Shifting responsibilities and recalibrating the team; and change of architecture required to enable integration of telehealth service delivery
Stern et al. 2012 Canada	To explore the experience and perceptions of family caregivers and palliative cancer patients with home telehealth	14 specialist nurses: sex NR, age NR, experience NR	Videophones, with optional remote monitoring of blood pressure, blood oxygen levels, and heart, lung and abdominal sounds	Mixed-methods case study; emphasizing qualitative methods	2 themes: enhanced access to care and usability of the home telehealth system. Overall tele-nurses felt that home telehealth enabled family caregiving, citing increased access to care, and patient and family caregiver reassurance. Pain management was the most common reason for initiating contact with the nurse, followed by emotional support. Concerns included lack of integration of services, inappropriate timing of the intervention and technical problems
Tieman et al., 2016 Australia	To assess the feasibility of a telehealth-based model of service provision for community based PC patients, carers and clinicians	HCPs made 121 ratings on conducting clinical assessments using videocalls compared to a phone call and 111 ratings comparing videocalls to face to face, sex NR, age NR, experience NR	Videoconferences between service staff and the patient or carer, virtual case conferences with the patient, family and HCPs, self-report assessment tools for the patient and carer, and remote activity monitoring.	Quantitative; following each video interaction with a client, the clinicians completed a brief assessment on the quality of the technology and the significance of the interaction	The nurses reported that videocalls were similar to or better/much better than phone calls or face-to-face contacts. Nurses indicated that technology was very effective in: reassuring the patient or carer; quick problem management; identification of problems; resolving issues that would have previously required a home visit; and sharing information with other health professionals. Issues with the volume of alerts generated, technical support required and the impact of service change were identified
van Gurp et al. 2015 The Netherlands	To investigate the practical and normative fit of weekly real-time audiovisual tele- consultations between home-based patients who require palliative care, their primary care physicians, and hospital-based PC specialists	12 hospital-based SPCT[9] clinicians (4 nurses, 8 physicians); sex NR, age NR, experience NR 18 PCP[10]; sex NR, age NR, experience NR	Weekly videoconferencing between a hospital-based SPCT and palliative care patients living at home using desktop computer or iPad. PCPs were invited to attend the VC at patient's home. If	Qualitative;. long-term direct observations, semi-structured interviews, open interviews	Videoconferencing was useful for diagnosis. Essential colors for diagnosis, small emotional cues, and physical indicators of decline were often not discernible in these images. SPCT clinicians did notice general physical progress or regression over time and/or mental states. Videoconferencing could be used to determine the patients' personalities and social contexts and to tend to the proxies and provide them with necessary counseling. SPCT clinicians avoided discussing sensitive, emotional topics with particularly vulnerable

Author, year, Country	Aim	Sample	Telehealth application	Design and methods	Results
			PCPs could not be present SPCT shared the content of VC with PCPs	following the observations	patients via VC because they did not feel sufficiently close to be able to comfort these patients
van Gurp et al. 2016 The Netherlands	To describe whether and how teleconsultation supports the integration of primary care, specialist PC, and patient perspectives and services and (2) how patients and (in)formal caregivers experience collaboration in a teleconsultation approach	12 hospital-based SPCT clinicians (4 nurses, 8 physicians); 6 males, age range 25-65 years, experience NR 17 PCP; 10 males, age range 25-65 years, experience NR	Weekly videoconferencing between a hospital-based SPCT and palliative care patients living at home using desktop computer or iPad. PCPs were invited to attend the videoconferencing at patient's home. If PCPs could not be present SPCT shared the content of videoconferencing with PCPs	Qualitative; long-term direct observations, in-depth interviews	Videoconferencing stimulated the integration of primary care and specialist palliative care by enabling bilateral conversations between a home-based patient and a hospital-based SPCT clinician/nurse, which were followed by backstage consultations between SPCT clinician and PCP. In case of cautious planning and physicians committing to information sharing, this backstage work led to better interprofessional understanding of one another's working contexts, to more practical and accurate multidisciplinary discussions, and to specialists being more approachable
Weck et al., 2019 Germany	To investigate HCPs experience with a video counseling system used to provide expert care for neurological outpatients in a palliative setting	Leading physicians at each study site (n=5), sex NR, age, NR, experience NR	Mobile technology for video counseling. When outpatient HCPs identify a problem, video counseling is initiated between patients, HCPs and the expert medical center	Mixed-methods; quantitative (technical quality of teleconsultation) and qualitative (semi-structured ethnographic interviews)	Telehealth was of sufficient quality to enable timely neurological assessments. Physicians reported improved patient satisfaction, access to specialized care and symptom awareness among HCPs. The visual component of video counseling was highlighted as a significant improvement. Connectivity issues, screen sizing and audio issues were reported. HCPs were able to troubleshoot problems
Whitten et al., 2001, USA	To present preliminary results from a bi-state telehospice project, on the use of videophones to supplement traditional care for terminally ill patients living in rural and urban areas. Specifically, the first-year	HCP and providers n= 47, sex NR, age NR, experience NR	The use of videophones by hospice patients and hospice providers as a supplement to regular care for terminally ill patients.	Mixed methods: surveys, nurses' notes and telephone interviews.	The results show a favorable preconception of the TH[11] equipment to be safe and effective by hospice providers, while hospice nurses were more ambivalent. The results from the first year of the project show a general increase and consistency of the number of patients serviced during the first year of the project. In sum, the HCP/provider surveys

Author, year, Country	Aim	Sample	Telehealth application	Design and methods	Results
	experiences aimed to offer insights on pre-perceptions, patient- and caregiver perceptions and satisfaction and the utilization of data from service providers and patients				reflected more barriers compared to the patient/caregiver surveys. HCP agree that telemedicine calls should be used for after hour's acute problems, showing some reluctance when considering using TH during normal working hours. HCP see a particular potential in using TH for emotional support of anxious patients/caregivers
Whitten et al., 2009 USA	To investigate why hospice nurses were slow to adopt videophones to care for their patients	25 hospice employees (18 were in a clinical position, 7 were non-clinical); sex NR, age NR, experience NR	Videophones, same as analogue telephone, but with a video screen	Qualitative; interviews, focus groups	39% reported no videophone training, despite the fact that every employee had received training. 4 staff members used a videophone with patients. HCPs overwhelmingly stated that they had the organizational resources necessary to use the videophone and that it was easy to operate. Despite initial enthusiasm, leaders in the hospice agency did not endorse the videophones for work, nor offer incentives for using the videophones or providing them to patients. It is important to note that videophone technology is not meant to replace face-to-face visits, but to supplement them and to provide an additional tool for the nurses

[1]Health care professionals; [2]Not reported; [3]Palliative care; [4]Specialized palliative care; [5]Amyotrophic lateral sclerosis; [6]Nonadoption, abandonment, scale-up, spread, and sustainability; [7]Home-based palliative care; [8]Remote home care; [9]Specialized palliative care teams; [10]Primary care physician; [11]Telehospice
